# Recent advances in IAP-based PROTACs (SNIPERs) as potential therapeutic agents

**DOI:** 10.1080/14756366.2022.2074414

**Published:** 2022-05-19

**Authors:** Chao Wang, Yujing Zhang, Lingyu Shi, Shanbo Yang, Jing Chang, Yingjie Zhong, Qian Li, Dongming Xing

**Affiliations:** a The Affiliated Hospital of Qingdao University, Qingdao University, Qingdao, China; b Cancer Institute, Qingdao University, Qingdao, China; c The Affiliated Cardiovascular Hospital of Qingdao University, Qingdao University, Qingdao, China; d School of Life Sciences, Tsinghua University, Beijing, China

**Keywords:** PROTACs, IAP, SNIPERs, degradation, promising treatment

## Abstract

Proteolytic targeting chimaeras (PROTACs) have been developed as an effective technology for targeted protein degradation. PROTACs are heterobifunctional molecules that can trigger the polyubiquitination of proteins of interest (POIs) by recruiting the ubiquitin-proteasome system, thereby inhibiting the intracellular level of POIs. To date, a variety of small-molecule PROTACs (CRBN, VHL, IAP, and MDM2-based PROTACs) have been developed. IAP-based PROTACs, also known as specific and nongenetic IAP-dependent protein erasers (SNIPERs), are used to degrade the target proteins closely related to diseases. Their structures consist of three parts, including target protein ligand, E3 ligase ligand, and the linker between them. So far, many SNIPERs have been extensively studied worldwide and have performed well in multiple diseases, especially cancer. In this review, we will present the most relevant advances in the field of SNIPERs and provide our perspective on the opportunities and challenges for SNIPERs to become therapeutic agents.

## 1. Introduction

Traditional small-molecule drugs have dramatically changed the face of diseases treatment in the last few decades. A variety of inhibitors, agonists, and antagonists have entered clinical use^1–3^. They are characterised by more acceptable pharmacokinetic properties and desirable oral bioavailabilities, as well as lower manufacturing costs. However, traditional small-molecule drugs still have many weaknesses that limit their application on non-pharmacological proteins such as transcription factors, scaffolding proteins, and non-enzymatic proteins. In terms of mode of action, traditional small-molecule drugs exert their effects by occupying active pocket sites, which requires high doses of administration to maintain activities. This increases the risk of off-targeting and leads to adverse effects. Moreover, refractory drug targets do not bind effectively to traditional small-molecule drugs. Furthermore, genetic mutations often lead to changes in protein conformation that can result in resistance to traditional small-molecule drugs. In addition, sustained inhibition of the target proteins may lead to compensatory overexpression of the proteins, which may greatly increase the risk of acquired drug resistances^4–6^. Therefore, it is of great importance to develop new technologies to address these issues.

**Figure 1.**
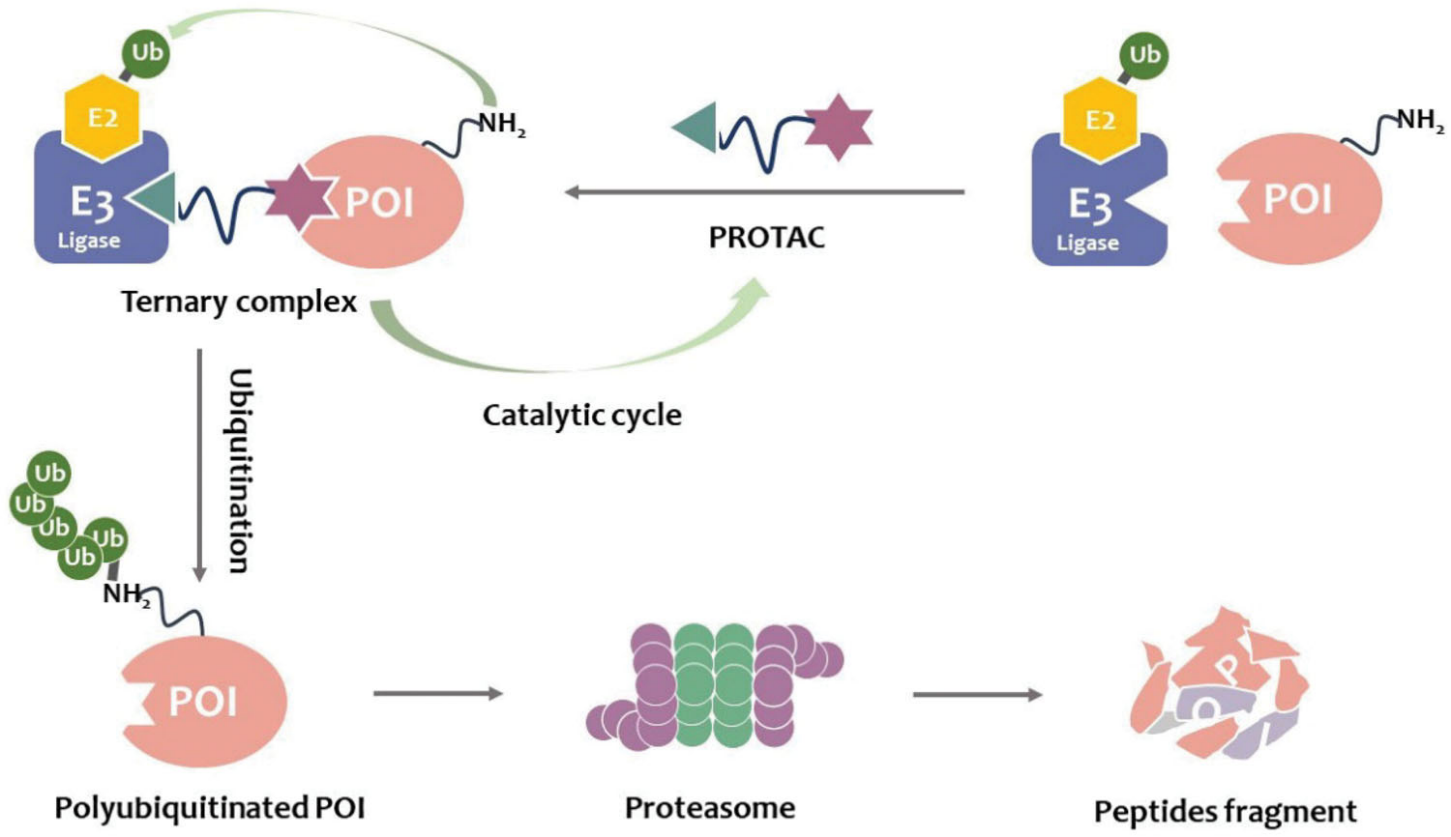
PROTACs-mediated degradation of target proteins through the UPS.

Alternatively, a new technology for targeting diseases-causing proteins is emerging. Proteolytic targeted chimaeras (PROTACs) are a new technique for targeted degradation of proteins of interest (POIs) that have attracted increasing research interest in recent years. PROTACs utilise ubiquitin-proteasome system (UPS) to degrade POIs^7–10^. During degradation, their E3 ligase ligands hijack the E3 ligase and label POIs with ubiquitin. PROTACs are fundamentally different from small-molecule drugs. Traditional small-molecule drugs bind specifically to the cavity of the target proteins in an occupation-driven manner. In contrast, PROTACs are event-driven and induce ubiquitination in a transient binding manner to eliminate the diseases-causing proteins. PROTACs themself are not degraded, they are recycled to facilitate ubiquitination and degradation of other target proteins (Figure 1). Importantly, this mode of action avoids a high level of drug administrations and the corresponding adverse effects. Moreover, to some extent, PROTACs can solve the problem of acquired drug resistances caused by POIs mutations and conventional inhibitors. Thus, PROTACs can be used to overcome the common drawbacks of traditional occupation-driven small-molecule drugs^11–13^.

**Figure 2.**
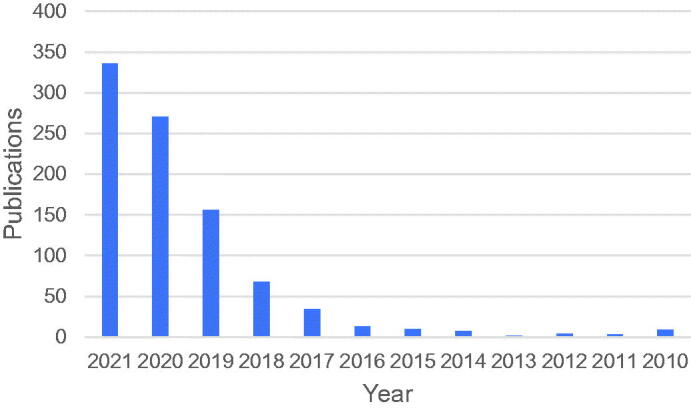
The number of publications on PROTACs/SNIPERs in PubMed (accessed on 12/13/2021).

The concept of PROTACs was proposed by Craig M. Crews and Raymond J. Deshaies in 2001^14^. The first-generation of PROTACs were heterofunctional molecules consisting of angiogenesis inhibitors and 10-amino acid phosphopeptides, which had the disadvantage of low cell permeability and stability in biological systems^15^. In order to solve the above issues, Craig M. Crews *et al.* developed the second-generation of PROTACs (small-molecule PROTACs) capable of successfully inducing androgen receptor (AR) degradation in 2008^16^. These PROTACs associated selective AR modulators with nutlin, recruiting the E3 ubiquitin ligase human homolog mouse double molecule 2 (MDM2). Recently, the third-generation of PROTACs with cereblon (CRBN), von Hippel-Lindau (VHL), and inhibitors of apoptosis (IAP) ligands have been developed rapidly, offering hope for the discovery and development of therapeutic agents with novel mechanisms and efficacy (Figure 2 and Table 1) ^18–21^.

**Table 1.** Overview of current PROTACs^17^.

Degraders	Description	Advantages	Disadvantages
CRBN-based PROTACs	Bifunctionals recruiting E3 CRBN; by far most common degrading mechanism used.	Wide target scope; compact warhead can allow oral exposure.	Narrow warhead SAR; heterogeneous responses across cells; high resistance potential; toxicological issues.
VHL-based PROTACs	Bifunctionals recruiting E3 VHL; second only to CRBN in breadth of reported use.	Bifunctionals recruiting E3 VHL; second only to CRBN in breadth of reported use.	High complexity of warheads limits oral exposure, CNS penetration, etc.
IAP-based PROTACs (SNIPERs)	Bifunctionals recruiting multiple E3s including cAIP1, cIAP2, and XIAP.	Parallel degrading pathways with potential to increase degradation efficiency.	Target scope not as well defined due to fewer reports; high warhead complexity limits druglike properties.
PROTACs based on other E3 ligases	Bifunctionals recruiting other E3 ligases, such as MDM2, RNF114, and DCAF15, 16.	Can offer novel profiles and novel chemical warheads.	Few data yet to assess potential; dependency on single ligase can cause scope and resistance issues.

**Figure 3.**
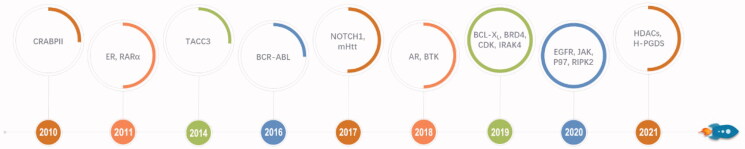
Timeline and major milestones for the development of SNIPERs.

IAP are a class of negative regulators for apoptosis that can inhibit cell apoptosis by inhibiting caspase^22–24^. IAP family consists of eight members, of which c-IAP1, c-IAP2, and XIAP are the most widely studied members of IAP^25^^,^^26^. IAP are often overexpressed in cancer cells and are closely related to poor prognoses, thus they are shown to be important targets for cancer therapy^27^^,^^28^. Apart from being the key targets for cancer therapy, IAP belong to the E3 ubiquitin ligase family. IAP-based PROTACs are also called specific and nongenetic IAP-dependent protein erasers (SNIPERs) ^29^^,^^30^. IAP inhibitors and their derivatives have been commonly used as E3 ligase ligands for SNIPERs. SNIPERs have been successfully employed in the degradation of different types of target proteins related to various diseases, including cancer, immune disorders, and neurodegenerative diseases. These target proteins mainly include AR, BCL-X_L_, BCR-ABL, BTK, BRD4, CDK, CRABPII, EGFR, ER, HDACs, H-PGDS, IRAK4, JAK, mHtt, NOTCH1, P97, PDE4, RARα, TACC3, etc.^29^^,^^31^^,^^32^. Therefore, SNIPERs are currently considered a promising approach for disease treatment. Figure 3 shows the milestones in the development of SNIPERs. In this review, we will summarise the most IAP E3 ligands and sum up the rapid progress from 2010 to 2021 in SNIPERs. We will also provide our perspective on the opportunities and challenges for SNIPERs to become therapeutic agents.

## 2. IAP ligands

**Figure 4.**
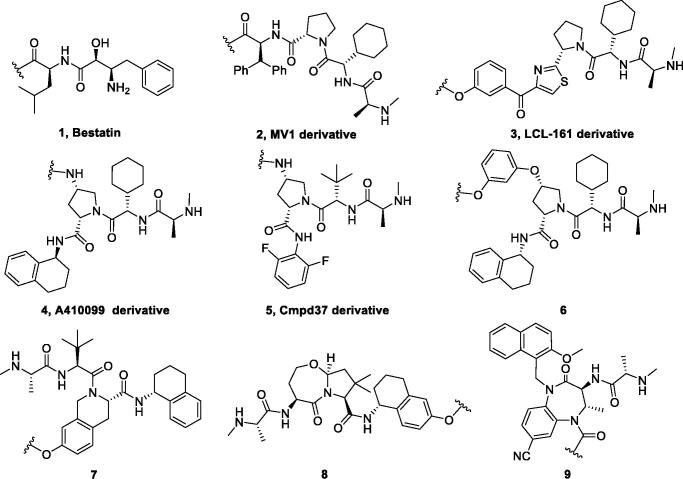
Chemical structures of small-molecular IAP ligands.

Because of IAP’s involvement in multiple malignancies, inhibitors of IAP represent an attractive strategy for tumour therapy, and many potent peptidomimetic antagonists have been developed^33–35^. Interaction between IAP antagonists and their targets results in the autoubiquitylation and proteasomal degradation of cIAP1. The methyl bestatin derivative was used as the cIAP1-binding ligand for the early reported degraders recruiting this E3 ligase^36^. The subsequent development of high-affinity IAP ligands and their incorporation into bifunctional molecules improved the efficiency of SNIPERs in comparison with early bestatin-based compounds. Structures of IAP ligands utilised in chimeric molecules are presented in Figure 4.

**Figure 5.**
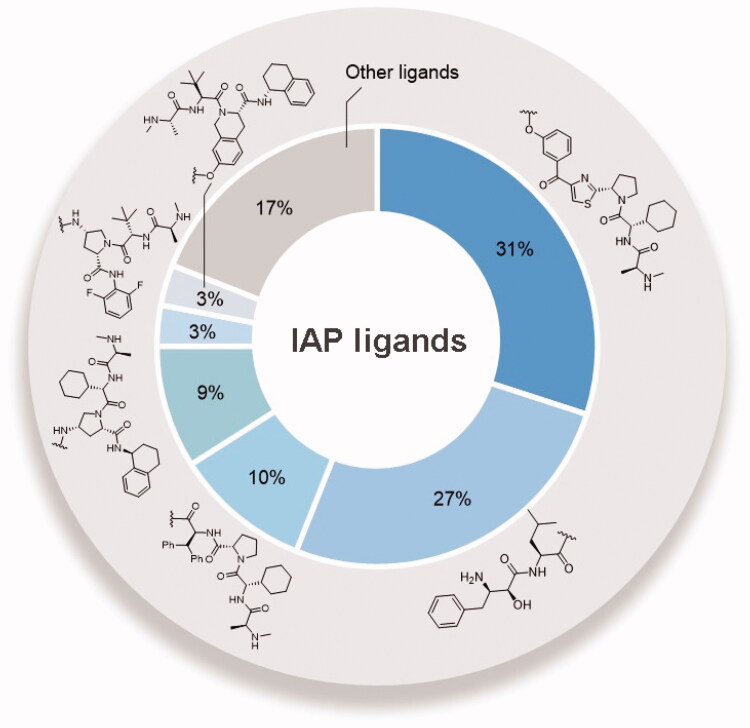
Frequency of IAP ligands used in SNIPERs.

Statistics were performed using data extracted from PubMed and PROTAC-DB to determine the frequency of various IAP ligands used in SNIPERs (Figure 5) ^37^^,^^38^. The LCL-161 derivative was the most commonly used, with approximately 31% of SNIPERs employing its structure. It was closely followed by bestatin, with lower occurrence of MV1 derivatives and IAP ligand 4 at 10% and 9%, respectively. Other IAP ligands were less common, with less than 3% representation.

## 3. Snipers for cancers

### 3.1. Targeting AR

The AR, a member of the nuclear hormone receptor superfamily, plays a vital role in the maintenance of male secondary sexual characteristics and development of the prostate gland. Disorder of the androgen receptor is the main driving force for prostate cancer (PC). AR antagonists, such as enzalutamide, apalutamide, and darolutamide, are effective for the treatment of PC. Unfortunately, patients treated with these AR antagonists ultimately develop drug resistances. In most tumours that are resistant to AR antagonists, the AR signalling continues to be functional and drives tumour growth and progression. Some of the major resistance mechanisms to AR antagonists include AR gene amplification and mutation and expression of AR variants^39^^,^^40^. Therefore, new therapeutic strategies to effectively target the AR signalling in tumours resistant to AR antagonists are urgently needed.

In 2018, Mikihiko Naito et al. developed a series of AR SNIPERs by linking AR antagonists with IAP ligands^41^. SNIPER-1 (Table 2) showed the ability to effectively degrade AR at 3 μM concentration. Moreover, SNIPER-1 could efficiently inhibit AR-mediated gene expression and suppress the proliferation of androgen-dependent PC cells. In addition, SNIPER-1 was found to be able to induce caspase activation and apoptosis in PC cells efficiently, which didn’t appear in the cells treated with AR antagonists. These results suggested that AR SNIPERs might serve as leads for an anticancer drug against PC that exhibit AR-dependent proliferation. AR SNIPERs are a promising strategy for the treatment of PC, further optimisation is necessary to develop more efficient AR degradation inducers for clinical use in the future.

**Table 2.** Representative SNIPER targeting AR.

Compound	Target protein	Structure	Reference
SNIPER-1	AR	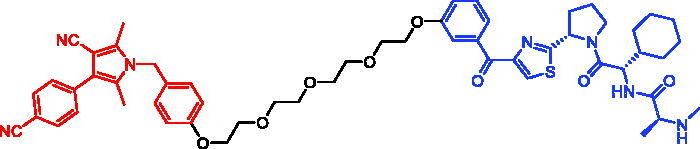	41

### 3.2. Targeting BCL-X_L_

B-cell lymphoma extra-large (BCL-X_L_) is one of the important proteins in the B-cell lymphoma 2 (BCL-2) family, which plays a pivotal role in controlling the life-cycle of cell *via* regulating the intrinsic apoptotic pathway. BCL-X_L_ is a well-validated cancer target. Inhibition of these BCL-2 family proteins with small molecules has been extensively investigated as a therapeutic strategy for cancers, resulting in the discovery of ABT263 (navitoclax, a BCL-2 and BCL-X_L_ dual inhibitor), ABT199 (venetoclax, a BCL-2 selective inhibitor), and several BCL-X_L_ and MCL-1 monoselective inhibitors as promising anticancer drug candidates. Currently, ABT199 is the only Food and Drug Administration (FDA)-approved antitumor agent targeting the BCL-2 family proteins. The use of BCL-X_L_ inhibitors in the clinic is largely limited by their on-target and dose-limiting platelet toxicity^42^^,^^43^. Targeting BCL-X_L_ *via* PROTACs/SNIPERs is a promising strategy in reducing BCL-X_L_ inhibition associated platelet toxicity.

In 2019, Guangrong Zheng et al. reported the potent BCL-X_L_ degraders that recruit VHL or CRBN E3 ligase. However, low protein expression or mutation of the responsible E3 ligase has been known to result in decreased protein degradation efficiency of the corresponding PROTACs. To overcome these mechanisms of resistance, PROTACs based on recruiting alternative E3 ligases could be generated. In 2020, they designed and synthesised a series of PROTACs that recruit IAP E3 ligases for BCL-X_L_ degradation^44^. Among those PROTACs, SNIPER-2 (Table 3) efficiently degraded BCL-X_L_ in malignant T-cell lymphoma cell line (MyLa 1929) while CRBN-based PROTACs that had high potency in other cancer cell lines showed compromised potency. Moreover, SNIPER-2 exhibited comparable cell killing in MyLa 1929 cells compared to the parent compound ABT-263, whereas the on-target platelet toxicity was significantly reduced, confirming that the therapeutic window could be improved by converting a BCL-X_L_ inhibitor to a BCL-X_L_ SNIPER. In addition, SNIPER-2 was able to induce powerful BCL-X_L_ degradation in multiple cancer cell lines, indicating that BCL-X_L_ SNIPERs had the potential of broad applications in cancer treatment.

**Table 3.** Representative SNIPER targeting BCL-X_L_.

Compound	Target protein	Structure	Reference
SNIPER-2	BCL-X_L_	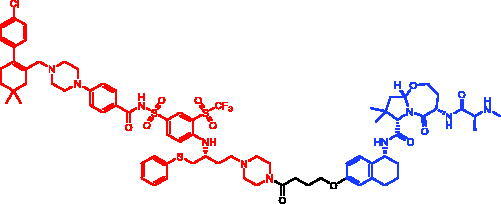	44

### 3.3. Targeting BCR-ABL

The oncogenic fusion protein BCR-ABL is the driving force of leukemogenesis in chronic myeloid leukaemia (CML). Several small-molecule tyrosine kinase inhibitors (TKI) have been developed for CML treatment. Imatinib is the first-generation TKI against the BCR-ABL, and it competitively binds to the ATP-binding site, resulting in the inhibition of cell proliferation. Although imatinib is currently used as the first-line therapy for CML patients, a significant number of patients develop resistance, usually due to point mutations in the tyrosine kinase domain of BCR-ABL protein. To overcome the imatinib resistance, second-generation TKI (e.g. dasatinib) have been developed. Such second-generation TKI are capable of saving most CML patients; however, drug resistances also occur against them^45^^,^^46^. Therefore, novel drug candidates with different mechanisms of action are required.

In 2016, Masaaki Kurihara et al. reported some SNIPERs targeting BCR-ABL by conjugating an imatinib derivative that binds to BCL-ABL and a bestatin that binds to cIAP1^47^. SNIPER-3 (Table 4), which contains a hexyl linker, and SNIPER-4, which possesses a decyl linker (Table 4), were found to induce degradation of BCR-ABL protein at 30 μM after incubation for 8 h and 24 h, respectively. SNIPER-3 and SNIPER-4 did not affect the mRNA levels of BCR-ABL, which suggested that they induce the degradation of BCR-ABL proteins with no effect on gene levels. Furthermore, both SNIPER-3 and SNIPER-4 significantly inhibited the growth of K562 cells. Therefore, the degradation of BCR-ABL by SNIPERs is one potential strategy for treating BCR-ABL driven chronic myelogenous leukaemia.

**Table 4.** Representative SNIPERs targeting BCR-ABL.

Compounds	Target protein	Structure	References
SNIPER-3	BCR-ABL	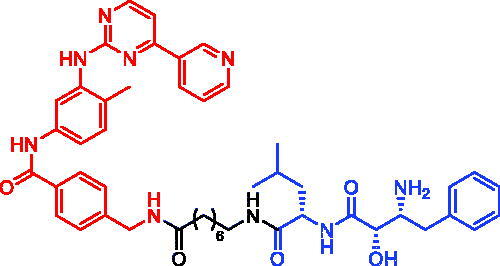	47
SNIPER-4	BCR-ABL	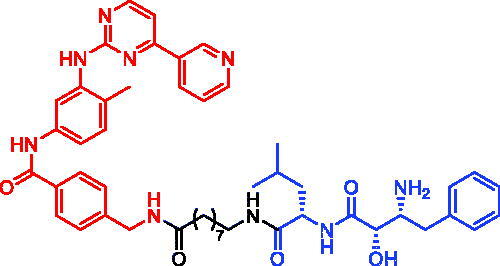	47
SNIPER-5	BCR-ABL	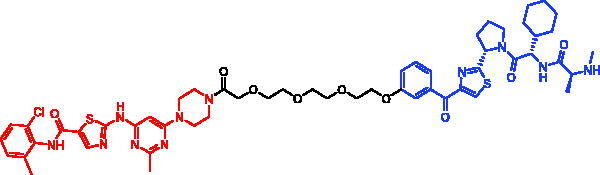	48
SNIPER-6	BCR-ABL	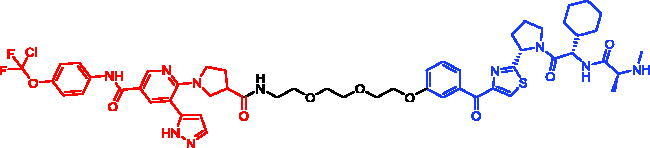	49

In 2017, Mikihiko Naito et al. developed some BCR-ABL SNIPERs by combination of different BCR-ABL inhibitors (e.g. imatinib, GNF5, and dasatinib) and IAP ligands (e.g. bestatin, MV1, and LCL161 derivatives)wwwwwwwwwwwwwwwwwwwwwwwwwwwwwwwwwwwwww^48^. The representative degrader, SNIPER-5 (Table 4), showed a potent activity to degrade the BCR-ABL in K562 cells at 10 μM through recruiting cIAP1 and XIAP. The maximum knockdown of BCR-ABL was observed at around 100 nM, when the cells were incubated with SNIPER-5 for 24 h. Furthermore, SNIPER-5 could inhibit the phosphorylation of BCR-ABL substrates. These results suggest that SNIPER-5 could be a candidate for a degradation-based novel anticancer drug against BCR-ABL-positive CML.

To further expand the capabilities of PROTACs/SNIPERs, Mikihiko Naito et al. synthesised a series of potential SNIPERs that binds allosterically to the nonorthosteric sites of oncogenic BCR-ABL proteins in the same year^49^. Among these compounds, SNIPER-6 (Table 4), showed desirable binding affinities against ABL1, cIAP1/2, and XIAP and efficiently induced degradation of BCR-ABL starting at 30 nM, with maximum activity at 100–300 nM. Therefore, SNIPER-6 that binds to the allosteric sites of target proteins may expand the capability of protein knockdown technology.

### 3.4. Targeting BRD4

Bromodomain-containing protein 4 (BRD4) is a protein that considered as a member of bromodomain and extra-terminal domain (BET) family, which serves as a reader to recognise the specific ε-N acetylated lysine residues on histone tails. The recognition and binding of the acetylated lysine sequences on histones of BRD4 could modulate multifarious downstream cell processes, including cell cycle, proliferation, and apoptosis^50^^,^^51^. After combining to acetylate histones, BRD4 interacts with the positive transcription elongation factor complex (P-TEFb) and thereby affects the activity of RNA polymerase II. The constitution of BRD4 includes two bromodomains (BD1 and BD2), N-terminal extra-terminal (NET) domain, and a C-terminal domain (CTD). Each bromodomain comprises the active acetyl-lysine-binding pocket which is formed by ZA loop and BC loop. Deregulation of BET protein activity, in particular BRD4, has been strongly linked to cancer and inflammatory diseases, making BET proteins attractive drug targets.

In 2019, Mikihiko Naito et al. produced two BRD4 degraders (SNIPER-7 and SNIPER-8, Table 5) by conjugation of the BET inhibitor (+)-JQ-1 to an IAP antagonist LCL-161 derivative^52^. Both SNIPERs were observed to rapidly reduce BRD4 protein levels within 6 h of treatment, with an optimal concentration of 0.1 µM. In addition to BRD4, SNIPER-7 could effectively reduce the protein levels of cIAP1 and XIAP within 6 h. They found that the degradation of cIAP1 and XIAP by SNIPER-7 was induced by different mechanisms. To investigate the mechanism of action, using a chemical biology-based approach, they developed two inactive SNIPERs, SNIPER-9 and SNIPER-10, incapable of degrading BRD4. SNIPER-9 contained an *N*-methylated LCL-161 derivative as the IAP ligand, which prevented it from binding IAP, and resulted in the abrogated degradation of cIAP1, XIAP, and BRD4. SNIPER-10, however, incorporated the enantiomer (−)-JQ-1 which was incapable of binding BRD4. SNIPER-10 degraded cIAP1 but lost the ability to degrade XIAP and BRD4. Furthermore, a mixture of the ligands, (+)-JQ-1 and LCL-161, induced the degradation of cIAP1, but not XIAP and BRD4. These results indicated that cIAP1 degradation is triggered by the binding of the IAP antagonist module to induce autoubiquitylation of cIAP1, whereas a ternary complex formation is required for the SNIPER-induced degradation of XIAP and BRD4.

**Table 5.** Representative SNIPERs targeting BRD4.

Compounds	Target protein	Structure	References
SNIPER-7	BRD4	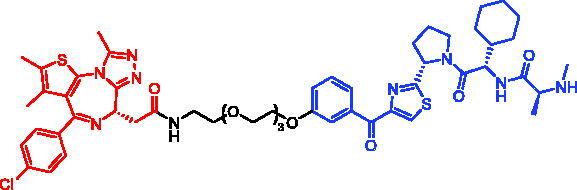	52
SNIPER-8	BRD4	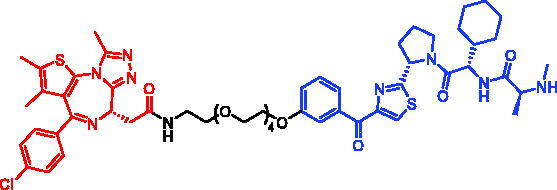	52
SNIPER-9	BRD4	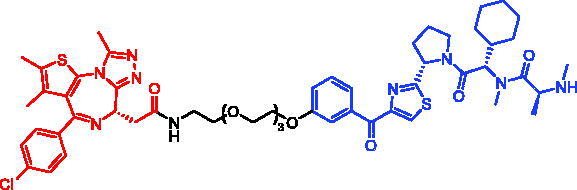	52
SNIPER-10	BRD4	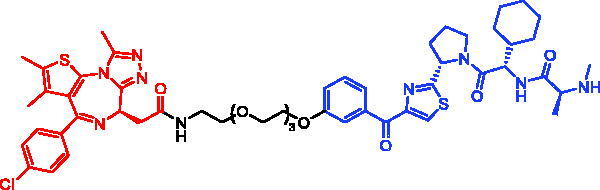	52

### 3.5. Targeting BTK

B-cell receptor (BCR) signalling is indispensable for B-cell’s adhesion, survival, and growth. As an important membrane proximal signal molecule in the BCR pathway, Bruton's tyrosine kinase (BTK) plays a key role in B cell activation and proliferation^53^^,^^54^. Inhibition of BTK kinase activity has been shown to be an important and practical approach for the treatment of non-Hodgkin's lymphoma (NHL). Ibrutinib is a class of covalent BTK inhibitors approved by the FDA for the treatment of several types of NHL. However, due to a missense mutation in BTK C481S, NHL patients have developed drug resistances after treatment with ibrutinib. Ibrutinib also lost the inhibitory effect on NHL tumour cell growth caused by the BTK C481S mutation^55^.

In 2018, a series of new BTK PROTACs/SNIPERs were designed and synthesised by Matthew F. Calabrese et al.^56^. The PROTACs/SNIPERs were developed through the conjugation of a BTK inhibitor and CRBN/VHL/IAP ligands. Among all PROTACs/SNIPERs, CRBN-based PROTACs had their abilities to induce BTK degradation. The authors found that BTK degradation was inefficient when either IAP or VHL was recruited instead of CRBN. Representative compound, SNIPER-11, was shown in Table 6.

**Table 6.** Representative SNIPERs targeting BTK.

Compounds	Target protein	Structure	References
SNIPER-11	BTK	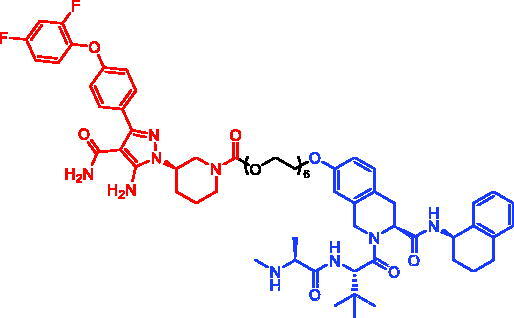	56
SNIPER-12	BTK	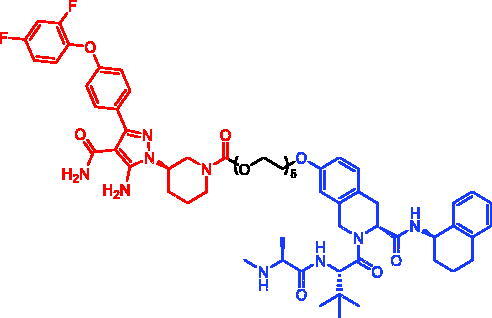	57
SNIPER-13	BTK	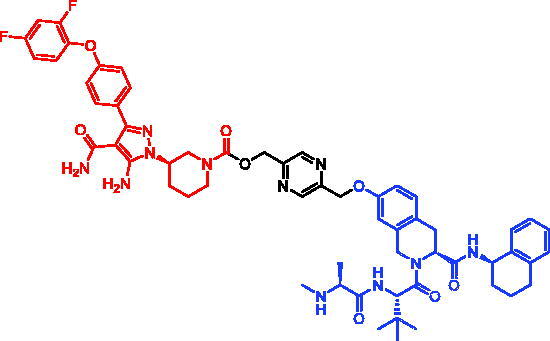	57

In 2020, Matthew F. Calabrese et al. reported a unique BTK degrader cIAP1 ternary complex crystal structure, providing a snapshot of three distinct binding poses between the target and E3 ligase^57^. Using both HSQC NMR and computational modelling, they designed and synthesised two new BTK SNIPERs (SNIPER-12 and SNIPER-13, Table 6) by linking aminopyrazole derivatives to IAP ligands. Treatment of THP-1 cells with SNIPER-12 led to a dose-dependent loss of BTK, with a half-maximum degradation concentration (DC_50_) of 182 ± 57 nM. This degradation was rescued by shortening the five-PEG linker to a non-permissive two-PEG linker (SNIPER-13) consistent with the protein degrader mechanism of action.

### 3.6. Targeting CDK

Cell cycle regulation is one of the vital and housekeeping activities in most types of the cells. Cyclin-CDK dual complexes are the major driving machinery for cell cycle progression. Among the CDKs, CDK6 plays a significant role in cell cycle entrance and is frequently overexpressed or hyperactivated in cancer samples^58^^,^^59^. Therefore, small molecule inhibitors of CDK6 have been officially approved or clinically tested to act against cancers including breast cancer, lymphoma, and multiple myeloma. However, overexpression of CDK6 induced by gene amplification or loss of FAT1 gene functions has been reported to be correlated to CDK4/6 inhibitor resistance in breast cancer cell lines and patient samples. Moreover, point mutation of CDK6 could possibly result in attenuation of drug binding affinity or hyperactivation of CDK6, though not yet reported in clinical samples^60^^,^^61^. Thus, there is an urgent need to develop a practical strategy against CDK6-centered malignancy.

In 2019, a focussed library of CDK6 degraders was developed and factors including linker length, spatial orientation, and binding affinity were systematically evaluated to help understand the match/mismatch between PROTACs/SNIPERs and targets and to deduce the best strategy for future design or optimisation of CDK6 degradation^62^. Yu Rao et al. found that CRBN-based PROTACs, instead of other tested E3 ligases, resulted in functional degraders. Representative IAP-based PROTACs, SNIPER-14 and SNIPER-15 (Table 7), were generated by linking E3 ligase IAP recruiter besatin to the FDA-approved CDK4/6 inhibitor palbociclib. Compared to CRBN-based PROTACs, SNIPER-14 and SNIPER-15 were not effective in degrading CDK6 protein and were not investigated more thoroughly by the authors.

**Table 7.** Representative SNIPERs targeting CDK.

Compounds	Target protein	Structure	References
SNIPER-14	CDK	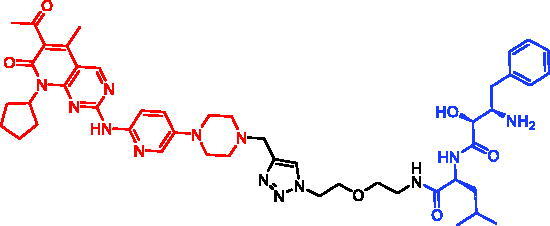	62
SNIPER-15	CDK	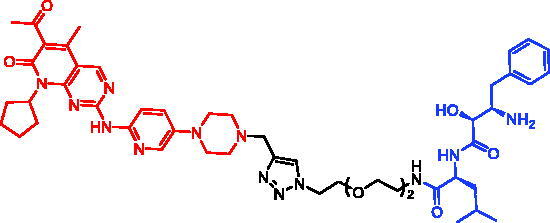	62
SNIPER-16	CDK	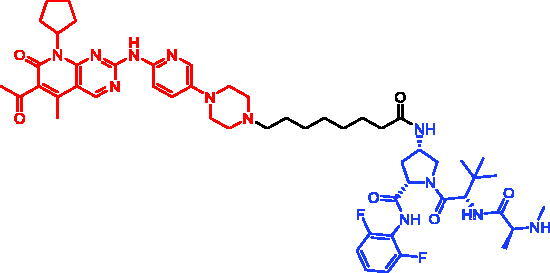	63
SNIPER-17	CDK	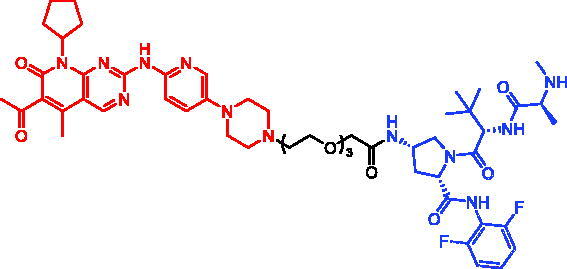	63
SNIPER-18	CDK	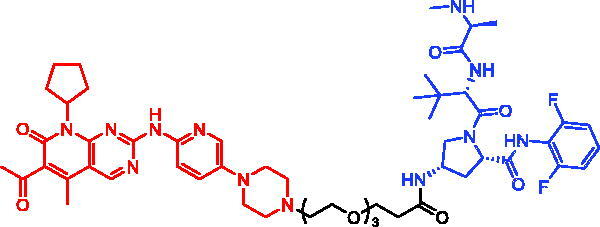	63
SNIPER-19	CDK	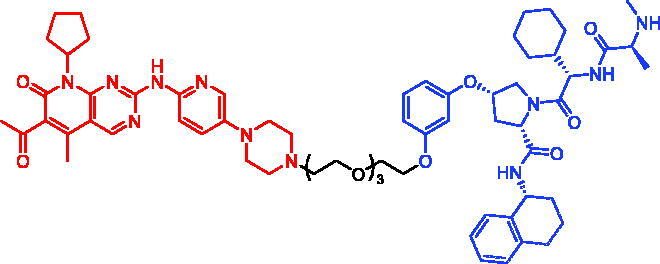	60
SNIPER-20	CDK	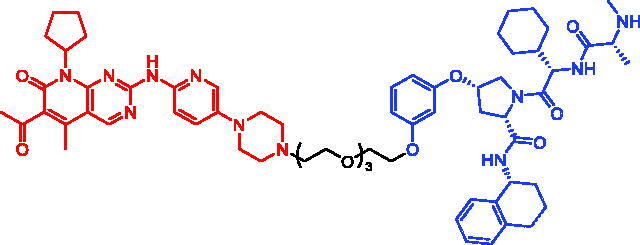	60

In 2020, Andrew B. Benowitz et al. developed some novel palbociclib-based CDK4/6 SNIPERs that incorporate a novel IAP-binder^63^. Following the preparation of SNIPER-16–SNIPER-18 (Table 7), they evaluated the degradation of CDK4 and CDK6 in Jurkat cells after 24 h compounds treatment in a dose–response manner using capillary electrophoresis combined with antibody labelling to measure protein levels. As expected, palbociclib did not cause any significant degradation of CDK4 or CDK6. However, they observed potent degradation of CDK4/6 for three SNIPERs.

In 2020, Jan Kronke et al. systematically explored the chemical space of CDK4/6 PROTACs by addressing E3 IAP ligase, and two novel multifunctional degraders (SNIPER-19 and SNIPER-20, Table 7), based on palbociclib and IAP ligands, were developed^60^. SNIPER-19 and SNIPER-20 could simultaneously and effectively degrade CDK4/6 with *D*_max_ of > 77% at 0.1 μM in MM.1S cells. Moreover, the new SNIPERs showed potent and long-lasting degrading activity in human and mouse cells and inhibited proliferation of several leukaemia, myeloma, and breast cancer cell lines. IAP-based PROTACs are an attractive approach for targeted degradation of CDK4/6 in cancer.

### 3.7. Targeting CRABP-II

Cellular retinoic acid binding protein II (CRABP-II) resides in cytoplasm and specifically binds to all-trans retinoic acid (ATRA), an endogenous ligand of retinoic acid receptors (RARs). CRABP-II is expressed in several cancers, including neuroblastoma and Wilms tumour. In particular, it is thought to play a role in cancer development in MycN-amplified neuroblastoma IMR-32 cells. It is reported that decrease of CRABP-II induced down-regulation of MycN, activation of caspase and growth inhibition of MycN-amplified neuroblastoma IMR-32 cells^18^^,^^64^.

In 2010, Yuichi Hashimoto et al. first reported a series of CRABP-II SNIPERs through connecting ATRA and bestatin *via* a linker, which could recruit cIAP1 and induce selective poly-ubiquitylation of CRABP-II to cause proteasomal degradation^18^. Among them, SNIPER-21 (Table 8) displayed the best potency, which could degrade CRABP-II in HT1080 cells at 1 μM, and showed a concentration-dependent trend. However, SNIPER-21 often induced cIAP1 autoubiquitination and degradation, which could be a concern for their use in therapy.

**Table 8.** Representative SNIPERs targeting CRABP-II.

Compounds	Target protein	Structure	References
SNIPER-21	CRABP-II	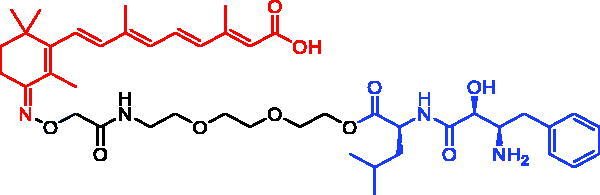	18
SNIPER-22	CRABP-II	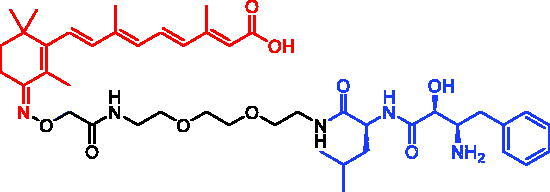	65
SNIPER-23	CRABP-II	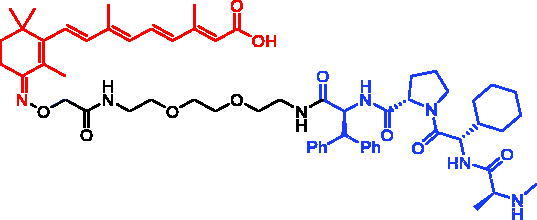	65

In 2012, Yuichi Hashimoto et al. designed and synthesised SNIPER-22 (Table 8), which contains bestatin as the E3 ligase ligand and induces selective degradation of CRABP-II but not of IAP^65^. They found that SNIPER-22 exerted an almost equivalent effect with SNIPER-21 in the aspect of degrading CRABP-II. Importantly, SNIPER-22 produced a more long-lasting degradation effect. However, SNIPER-22 was less effective than SNIPER-21 in inhibiting the proliferation of neuroblastoma IMR32 cells. Subsequently, taking IAP inhibitor MV-1 as E3 ligase ligand, they developed SNIPER-23 (Table 8), which was nearly 10 times more potent than SNIPER-21 in the area of degrading CRABP-II and cIAP1, and also could effectively inhibit the proliferation of IMR32 cells.

### 3.8. Targeting EGFR

Epidermal growth factor receptor (EGFR) is a glycoprotein with tyrosine kinase activity that is a major member of the erythroblastosis oncogene B (ErbB) family. EGFR is involved in tumour cell proliferation, angiogenesis, tumour invasion, metastasis, and inhibition of apoptosis. The overexpression of EGFR plays an important role in the progression of malignant tumours, such as glioblastoma, head and neck cancer, NSCLC, pancreatic cancer, breast cancer, and so on. Through decades of development, three generations of EGFR inhibitors have emerged. Despite great therapeutic successes, the clinical application of three generations of EGFR inhibitors inevitably leads to acquired drug resistances, which has presented a new challenge of treating cancers^66–68^.

In 2020, a series of selective EGFR^L858R/T790M^ mutant PROTACs/SNIPERs were designed and synthesised based on pyrido [2,3-d]pyrimidin-7-one selective EGFR^L858R/T790M^ inhibitor XTF-262^69^. The VHL-based PROTACs effectively and selectively degraded EGFR^L858R/T790M^ with a DC_50_ value of 5.9 nM. In contrast to VHL-based PROTACs, IAP-based PROTAC (SNIPER-24, Table 9) was unable to degrade EGFR^L858R/T790M^ protein, which was not overly described by the authors.

**Table 9.** Representative SNIPER targeting EGFR.

Compound	Target protein	Structure	Reference
SNIPER-24	EGFR	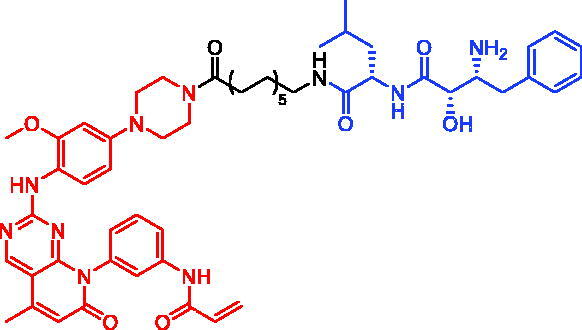	69

### 3.9. Targeting ER

Breast cancer (BC) is one of the most common malignancies in women worldwide. Oestrogen receptors+ (ER+) breast cancer occurs in approximately 80% of newly diagnosed breast cancer cases. As members of the nuclear receptor family, oestrogen receptors ERα and ERβ are transcription factors regulating gene expression and mediating the biological effects of the oestrogen. Both ERα and ERβ are widely expressed in different tissues, and ERα is considered to be the major mediator which transduces the oestrogen signalling in the female reproductive tract and mammary glands. ERα has therefore been pursued as a promising therapeutic target in multiple pathological settings, particularly in cancer and osteoporosis^70^^,^^71^.

In 2011, Yuichi Hashimoto et al. reported SNIPER-25 (Table 10) targeting ER by linking oestrone ligand and IAP ligand (bestatin) within a single molecule^72^. They found that SNIPER-25 at 1 μM significantly decreased ERα level in human breast cancer cell MCF-7.

**Table 10.** Representative SNIPERs targeting ER.

Compounds	Target protein	Structure	References
SNIPER-25	ER	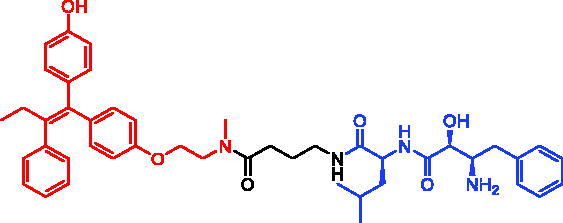	72
SNIPER-26	ER	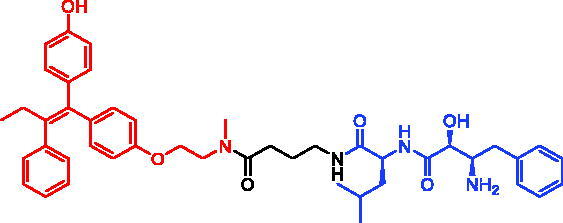	73
SNIPER-27	ER	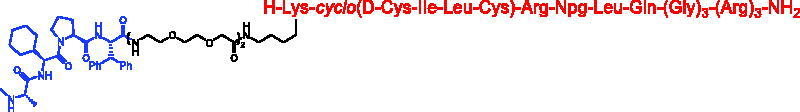	74
SNIPER-28	ER	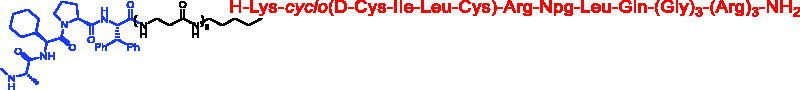	74
SNIPER-29	ER	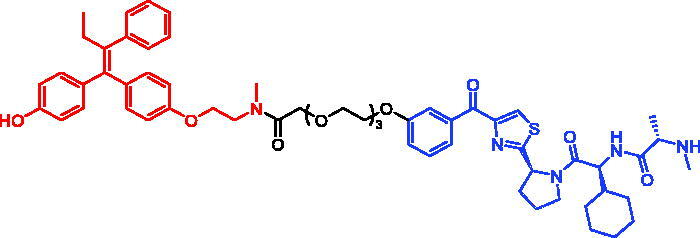	75
SNIPER-30	ER	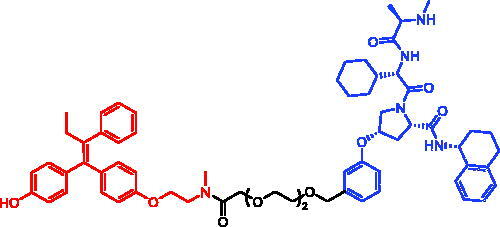	76
SNIPER-31	ER	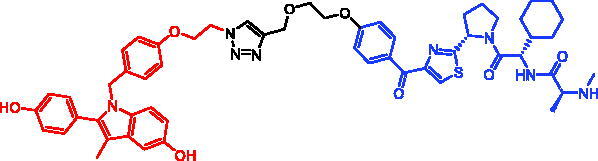	77
SNIPER-32	ER	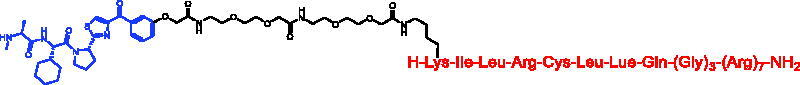	78
SNIPER-33	ER	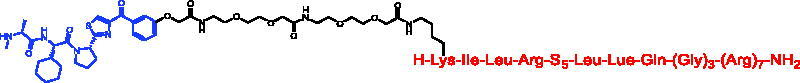	78

In 2012, Masaaki Kurihara et al. also synthesised three ERα SNIPERs by means of connecting 4-hydroxytamoxifen (4-OHT) with bestatin^73^. Among them, SNIPER-26 (Table 10) was effective in reducing ERα levels even at low concentrations of 10 μM. Moreover, SNIPER-26 was found to be able to induce the production of reactive oxygen species (ROS) in MCF-7 cells and then lead to cell death. In addition, the down-regulation of ERa by SNIPER-26 was also observed in breast cancer cell T47D^73^.

PERM3 is a peptide analogue of Steroid Receptor Coactivator 1 (SRC-1) that reacts with the ER surface R7, a fragment of heptarginine, is capable of improving the permeability of PERM3. In 2016, Masaaki Kurihara *et al.* discovered SNIPER-27 (Table 10) and SNIPER-28 (Table 10) based on PERM3-R7^74^. SNIPER-27 could decrease the levels of ERα and cIAP1 in a concentration-dependent manner, but toxic effects began to appear when the concentration was more than 6 μM in MCF-7 cells. The ability of SNIPER-28 for degrading ERα was slightly weaker than that of SNIPER-27, but toxic effects appeared at concentration of 20 μM.

In 2017, Mikihiko Naito et al. disclosed some SNIPERs. These compounds were developed by ligating 4-OHT with different IAP-binding compounds (bestatin, MV1, and LCL161) ^75^. However, bestatin-based SNIPER could only induce ERα degradation at 10 μM or higher. Then they developed MV1-based SNIPER, in which the IAP antagonist MV1 was substituted for bestatin. It successfully improved ERα degradation activity. They further developed SNIPER-29 (Table 10), with higher degradation activity, by using a derivative of LCL161. Unlike SNIPERs targeting ERα mentioned above, SNIPER-29 recruited XIAP instead of cIAP1 to ubiquitinate ERα for degradation. SNIPER-29 at 3 nM began to show degradation activity, and the best effect appeared at concentration of 100 nM. In MCF-7 tumour xenotransplantation mouse model, SNIPER-29 could significantly inhibit tumour growth, and displayed no obvious toxic side effects.

In 2018, they continued to develop a series of ERα SNIPERs using other reported IAP inhibitors^76^. Representative compound, SNIPER-30 (Table 10), was more effective than SNIPER-29 in inducing ERα degradation and apoptosis of breast cancer cell MCF-7. In addition, its ability of degrading ERα was better than that of SNIPER-29 in MCF-7 xenotransplantation mouse model.

In 2020, utilising DECL platform, Shilpi Arora et al. described some PROTACs/SNIPERs targeting ERα^77^. The VHL-based PROTACs effectively degraded ERα in ER + cells. They also tested VHL-based PROTACs in normal immortalised breast cells and observed no off-target effects. These PROTACs also demonstrated suitable properties for *in vivo* applications and were efficacious in an ERα-dependent xenograft model. Compared with VHL-based PROTACs, IAP-based PROTAC (SNIPER-31, Table 10) degraded ERα proteins with low efficiency, which had not been studied much by the authors.

In 2021, Yosuke Demizu et al. succeeded in developing the stapled peptide stPERML-R7, which was based on the ERα-binding peptide PERML and composed of natural amino acids^78^. stPERML-R7, which included a hepta-arginine motif and a hydrocarbon stapling moiety, showed increased α-helicity and similar binding affinity towards ERα when compared with those of the parent peptide PERML. On this basis, they used stPERML-R7 to develop a peptide-based degrader LCL-stPERML-R7 (SNIPER-32, Table 10) targeting ERα. The chimeric peptide SNIPER-32 induced sustained degradation of ERα and potently inhibited ERα-mediated transcription more effectively than the unstapled chimaera LCL-PERML-R7 (SNIPER-33, Table 10). These results suggested that a stapled structure was effective in maintaining the intracellular activity of peptide-based degraders.

### 3.10. Targeting HDACs

Histone deacetylases (HDACs) can catalyse the removal of acetyl group from lysine residues of histones or some non-histone proteins, therefore playing an essential role in the regulation of gene expression and protein function. Studies have shown that HDACs are closely associated with tumorigenesis and progression. To date, only five HDAC inhibitors have been approved as anticancer agents^79^^,^^80^. Therefore, more drugs targeting HDACs are eager to be discovered.

In 2021, three bestatin-based hydroxamic acids (SNIPER-34, SNIPER-35, and SNIPER-36, Table 11) were designed and synthesised by Yingjie Zhang et al.^81^. Those SNIPERs could work as HDACs degraders by recruiting cIAP1 E3 ubiquitin ligase. Among them, SNIPER-34 exhibited comparable even more potent inhibitory activity against HDAC1, HDAC6, and HDAC8 relative to the approved HDACs inhibitor SAHA. It is worth noting that although SNIPER-34 could not lead to intracellular HDACs degradation after 6 h of treatment, it could dramatically decrease the intracellular levels of HDAC1, HDAC6, and HDAC8 after 24 h of treatment. Moreover, all three SNIPERs exhibited more potent aminopeptidase N (APN, CD13) inhibitory activities than the approved APN inhibitor bestatin, which translated to their superior anti-angiogenic activities.

**Table 11.** Representative SNIPERs targeting HDAC.

Compounds	Target protein	Structure	References
SNIPER-34	HDACs	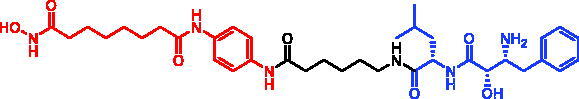	81
SNIPER-35	HDACs	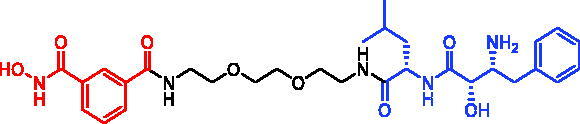	81
SNIPER-36	HDACs	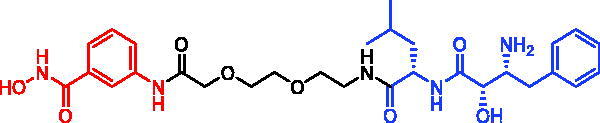	81

### 3.11. Targeting JAK

Janus kinases (JAKs) are a family of non-receptor tyrosine kinases, composed of four members, JAK1, JAK2, JAK3, and TYK2. JAKs are involved in different inflammatory and autoimmune diseases, as well as in malignancies, through the activation of the JAK/STAT signalling pathway. Furthermore, the V617F mutation in JAK2 was identified in patients affected by myeloproliferative neoplasms. This knowledge prompted researchers from academia and pharmaceutical companies to investigate this field in order to discover small molecule JAK inhibitors and degraders^82^^,^^83^.

In 2020, Rishi R. Shah et al. successfully described a set of novel SNIPERs targeting the JAK proteins by conjugating JAK inhibitor Quinoxaline with an IAP ligand^84^. SNIPER-37 was capable of inducing JAK1 and JAK2 degradation. At the highest concentration (5 µM) tested, where SNIPER-37 (Table 12) could induce JAK1 degradation by 48% and JAK2 degradation by 65%. The authors found that PROTACs bearing an IAP ligand induced significantly more JAK degradation over VHL- and CRBN-based PROTACs. In addition, the mechanism of action of the JAK SNIPERs was elucidated, and it was confirmed that JAK degradation was both IAP- and proteasome-dependent.

**Table 12.** Representative SNIPER targeting JAK.

Compounds	Target protein	Structure	Reference
SNIPER-37	JAK	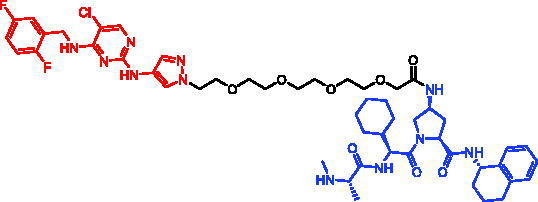	84

### 3.12. Targeting NOTCH1

Oncogenic transcriptional factor NOTCH1 plays a critical role in several biological functions such as cellular differentiation, proliferation, and death. Activated NOTCH1 is closely related to the initiation and development of some cancers. Ligands that bind to the extracellular domain of NOTCH receptors induce their proteolytic cleavage catalysed by ADAM family metalloproteinase and g-secretase, leading to the cytoplasmic release of the intracellular domain of NOTCH1 (ICN1). ICN1 translocates to the nucleus and regulates the activity of CSL, a DNA-binding transcription factor, together with MAML. ICN1 interacts with CSL, which forms a shallow groove along the surface of the two proteins that acts as a binding surface for the co-activator MAML1^85^^,^^86^.

In 2017, Mikihiko Naito et al. reported peptide-based SNIPERs for NOTCH1 degradation by conjugating MAML1 (21–26) or SAHM1 to MV-1^85^. Western blot showed that representative compound, SNIPER-38 (Table 13), effectively reduced NOTCH1 protein level in a dose-dependent manner, which was more effective than other SNIPERs. Moreover, after incubation with MOLT-4 human T-ALL cells within 2 h, SNIPER-38 also rapidly degraded the protein levels of c-MYC (a downstream target of NOTCH1) in a time-dependent manner. The above results indicate that the conjugation of MV1 and stapled peptides *via* an appropriate linker makes it feasible to develop potent inducers for NOTCH1 protein degradation.

**Table 13.** Representative SNIPER targeting NOTCH1.

Compounds	Target protein	Structure	Reference
SNIPER-38	NOTCH1	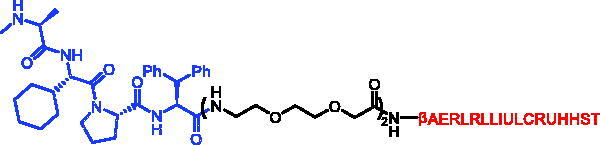	85

### 3.13. Targeting P97

The AAA + ATPase, P97, also referred to as VCP, plays an essential role in cellular homeostasis by regulating endoplasmic reticulum-associated degradation (ERAD), mitochondrial-associated degradation (MAD), chromatin-associated degradation, autophagy, and endosomal trafficking. Mutations in P97 have been linked to several neuro-degenerative diseases, and overexpression of wild type P97 is observed in numerous cancers. Furthermore, P97 activity has been shown to be essential for the replication of certain viruses, including poliovirus, herpes simplex virus (HSV), cytomegalovirus (CMV), and influenza. Taken together, these observations highlight the potential for targeting P97 as a therapeutic approach in neurodegeneration, cancer, and certain infectious diseases^87^^,^^88^. It is unclear what factors determine whether degradation of a ubiquitinated protein occurs in a P97-dependent or P97-independent manner. The ability to induce rapid polyubiquitination of large numbers of kinases with multitargeted degraders provided an opportunity to examine whether degradation of protein kinases is P97-dependent and if this dependency changes with differences in recruited E3 ligase.

To assess P97 dependence across the kinome, Eric S. Fischer et al. reported multiple P97 PROTACs/SNIPERs (e.g. SNIPER-39, Table 14) by tethering different E3 ligase ligands (CRBN, VHL, or IAP) with the P97 inhibitor GNF-7 in 2020^89^. Analysis of protein expression of the top kinase hits from each experiment revealed that the proposed P97 dependence was independent of the ligase responsible for mediating ubiquitination.

**Table 14.** Representative SNIPER targeting P97.

Compounds	Target protein	Structure	Reference
SNIPER-39	P97	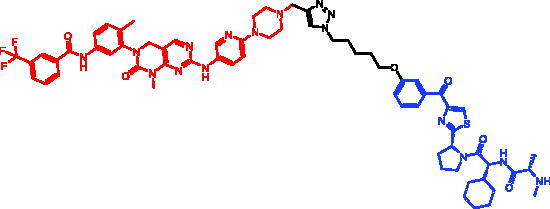	89

### 3.14. Targeting TACC3

Transforming acidic coiled-coil-3 (TACC3) belongs to spindleregulatory protein family. During mitosis, TACC3 localises to the mitotic spindle and plays a key role in spindle assembly, chromosome function, and mitotic progression. TACC3 was found to be overexpressed in a variety of human cancers, including ovarian cancer, breast cancer, squamous cell carcinoma, and lymphoma. Therefore, TACC3 is a target that suitable for anti-cancer drug discovery, which has attracted increasing attention from researchers, recently^90^^,^^91^.

In 2014, Mikihiko Naito et al. developed SNIPER-40 (Table 15) targeting TACC3 protein by connecting KHS101 to bestatin, which induced proteasomal degradation of the TACC3 proteins in HT080 cells after treated for 6 h at 30 μM and 24 h at 10 μM^92^. SNIPER-40 at ≥ 10 μM efficiently killed HT1080 and MCF7 cells after treatment for 48 h. SNIPER-40 selectively killed cancer cells that aberrantly express large amount of TACC3 proteins, but exerted no effect on normal cells. Therefore, targeting TACC3 for degradation by SNIPERs offers a new option for cancer therapy.

**Table 15.** Representative SNIPER targeting TACC3.

Compounds	Target protein	Structure	Reference
SNIPER-40	TACC3	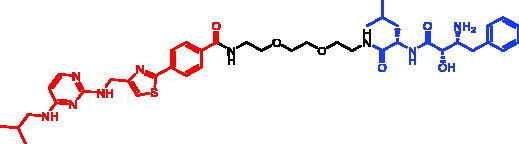	92

## 4. Snipers for immune diseases

### 4.1. Targeting H-PGDS

Overproduction of PGD2 is related to a variety of diseases, including allergic diseases, physiological sleep, and Duchenne muscular dystrophy. H-PGDS is one of the potential therapeutic targets for such diseases. *In vivo* studies have demonstrated that H-PGDS inhibition is effective in the treatment of allergic inflammation. To date, several types of H-PGDS inhibitors have been developed as therapies for allergic and inflammatory responses. However, the advancement of these inhibitors into clinical studies has not been satisfactory. Thus, the development of novel agents for clinical investigation with modes of action other than H-PGDS inhibition is required^93^^,^^94^.

In 2021, Yosuke Demizu et al. successfully developed some PROTACs/SNIPERs of the H-PGDS protein by conjugating H-PGDS ligand (TFC-007) to CRBN ligand (pomalidomide) or IAP ligand (LCL161 derivative)^95^. CRBN-based PROTACs effectively induced the selective degradation of H-PGDS protein *via* the UPS and showed sustained suppression of PGD2 production. CRBN-based PROTACs with a new mechanism of action were expected to be as effective or more effective than conventional inhibitors and might allow a reduction in the number of doses for the treatment of chronic inflammation. Compared to CRBN-based PROTACs, protein reduction activity for IAP-based PROTAC (SNIPER-41, Table 16) was not observed in MEG-01s cells expressing H-PGDS protein.

**Table 16.** Representative SNIPER targeting H-PGDS.

Compounds	Target protein	Structure	Reference
SNIPER-41	H-PGDS	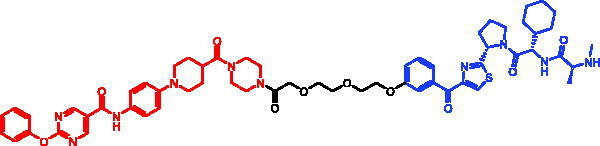	95

### 4.2. Targeting IRAK4

Interleukin-1 receptor-associated kinase 4 (IRAK4) belongs to a family of four kinases (IRAK4, IRAK1, nterleukin-1 receptor-associated kinase 4 (IRAK4) IRAK2, and IRAK-M). IRAK4 is a serine/threonine kinase that is involved in transduction pathways stimulated by the Tolllike receptors (TLRs) and the interleukin-1 (IL-1) family of receptors. Recognition of foreign pathogens and inflammatory signals by these receptors promotes IRAK4 binding to the adapter protein myeloid differentiation primary response gene (88) (MyD88) resulting in IRAK4 activation that in turn leads to the production of pro-inflammatory cytokines *via* the NFκβ pathway. IRAK4 deficiency or loss of function has been reported to increase susceptibility to several pathogens, while kinase activation has been linked with various autoimmune diseases such as systemic lupus erythematosus, psoriasis, rheumatoid arthritis, and cancer^96^^,^^97^.

In 2019, Niall A. Anderson et al. reported many PROTACs/SNIPERs targeting IRAK4 protein by using different E3 ligase ligands^98^. IAP-based PROTACs (SNIPER-42 and SNIPER-43, Table 17) were not able to degrade IRAK4 protein. The authors believed that there were many potential reasons why SNIPERs were not found to degrade IRAK4. For example, the linker may be the wrong length to facilitate efficient ternary complex formation. The orientation of the protein-IAP E3 ligase ternary complex may also not be able to promote efficient ubiquitin transfer onto an IRAK4 surface lysine residue. Finally, even though these compounds may bind to the protein, this does not always translate into degradation.

**Table 17.** Representative SNIPERs targeting IRAK4.

Compounds	Target protein	Structure	References
SNIPER-42	IRAK4	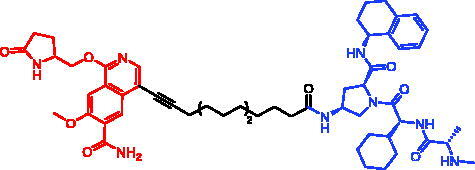	98
SNIPER-43	IRAK4	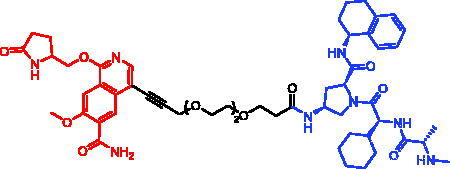	98

### 4.3. Targeting RIPK2

Receptor-interacting serine/threonine protein kinase 2 (RIPK2) is activated following intracellular recognition of the bacterial peptidoglycan fragments d-glutamyl-*meso*-diaminopimelic acid (iE-DAP) and muramyl dipeptide (MDP) by the pattern recognition receptors NOD1 and NOD2, respectively. Activation of RIPK2 results in its autophosphorylation and K-63 linked polyubiquitination which promotes recruitment of MAPK and NF-KB signalling scaffolds and leads to transcriptional activation of multiple inflammatory cytokine genes. Dysregulation of this pathway has been implicated in several diseases such as inflammatory bowel disease, severe pulmonary sarcoidosis, and multiple sclerosis^99^^,^^100^.

In 2020, John D. Harling et al. reported some IAP-based RIPK2 SNIPERs utilising an RIPK2 binder (aminobenzothiazole-quinoline)^101^. All three PROTACs potently degraded RIPK2 in a concentration-dependent manner. SNIPER-44 (Table 18) was found to have a log half-maximal degradation concentration (pDC_50_) value of 9.4 ± 0.1 and was more potent than either the equivalent VHL-based PROTAC (pDC_50_ = 8.7 ± 0.1) or the CRBN-based PROTAC (pDC_50_ = 8.6 ± 0.4). To obtain SNIPERs with better physicochemical properties and *in vivo* efficacies, the authors designed and synthesised new SNIPER-45 (Table 18) and SNIPER-46 (Table 18). SNIPER-46 contained an additional methylene spacer at the junction of the RIPK2 binding moiety and the linker, as well as a modified IAP binder. SNIPER-46 produced a concentration- and time-dependent decrease in RIPK2 protein levels in human PBMCs such that the pDC_50_ and *D*_max_ increased with longer incubation times, and a pDC_50_ of 9.4 ± 0.2 and *D*_max_ of 94.3 ± 3.2% was determined for 6 following 24 h incubation. SNIPER-46 completely inhibited TNFα, IL1β, IL-6, and IL-10 release following L18-MDP-stimulation with pIC_50_ values >8.8, but only partially inhibited IL-8 release (pIC_50_ = 8.5 and *I*_max_ ∼50%). SNIPER-46 was equally effective at inhibiting spontaneous cytokine release across CD and UC patient biopsy samples with IC_50_ values of ∼1–3 nM. RIPK2 protein levels were also assessed by western blot upon treatment of the CD and UC donor colon tissue with SNIPER-46.

**Table 18.** Representative SNIPERs targeting RIPK2.

Compounds	Target protein	Structure	References
SNIPER-44	RIPK2	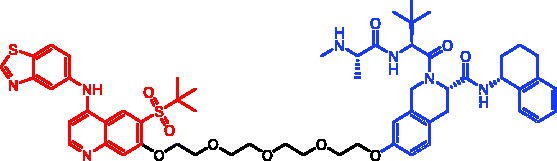	101
SNIPER-45	RIPK2	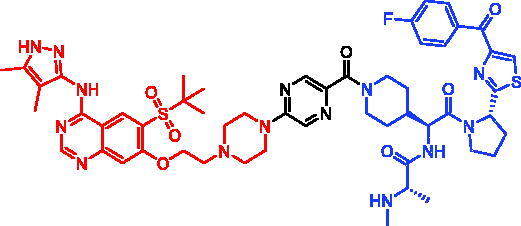	101
SNIPER-46	RIPK2	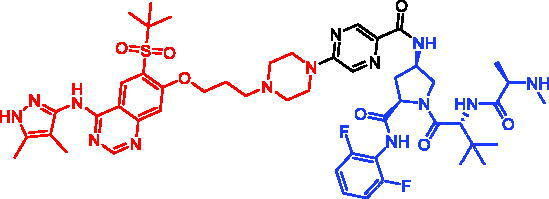	101
SNIPER-47	RIPK2	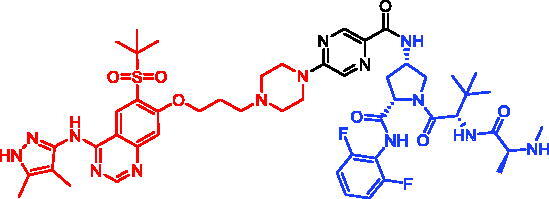	102

In 2021, John D. Harling et al. described the optimisation of a series of IAP-based PROTACs targeting RIPK2^102^. They focussed on reducing the lipophilicity of the early lead which resulted in the identification of analogues with improved solubility and increased human and rat microsomal stability. Representative compound, SNIPER-47 (Table 18), represented a highly potent and selective RIPK2 PROTAC. SNIPER-47, with attractive pharmacokinetic profiles, possessed the best overall profile with good solubility, potent degradation of RIPK2, and associated inhibition of TNFα release. It utilised a novel IAP binder which has reduced cIAP1 binding potency and increased XIAP BIR2 binding potency compared to most published SMAC mimetics. This allowed degradation of RIPK2 in the absence of cIAP1 autoubiquitination over a range of concentrations.

## 5. Snipers for neurodegenerative diseases

### 5.1. Targeting mHtt

Huntington’s disease (HD), an autosomal dominant neurodegenerative disorder, is caused by aggregation of mutant huntingtin (mHtt). Thus, removal of toxic mHtt may be an effective therapeutic approach for HD. Various approaches targeting the toxic mHtt aggregates have been developed and reported, including small molecules. Although many efforts have been made, effective clinical treatment is yet not available, owing to the modes of action of the chemical modulators for aggregation still remaining unclear. Additionally, biological approaches show several disadvantages, such as poor metabolic stability and selectivity, and low permeability^103^.

In 2017, Minoru Ishikawa et al. successfully achieved the targeting of mHtt protein aggregation with small-molecule SNIPERs. They designed and synthesised SNIPER-48 (Table 19) and SNIPER-49 (Table 19) by connecting BTA and PDB to bestatin *via* proper linkers, respectively^104^. Western bolt results disclosed that SNIPER-48 and SNIPER-49 at 10 μM could effectively reduce mHtt protein in two fibroblasts in a dose-dependent manner. Results of biological activity suggested that SNIPER-48 and SNIPER-49 worked effectively as down-regulators of mHtt in HD patients’ fibroblasts and were expected to be promising candidates for treatment of patients with HD. In conclusion, the authors described SNIPERs that can degrade mHtt *in vitro*. The authors didn’t describe much about the druglike properties of SNIPERs molecules, such as membrane permeability and metabolic stability.

**Table 19.** Representative SNIPERs targeting mHtt.

Compounds	Target protein	Structure	References
SNIPER-48	mHtt	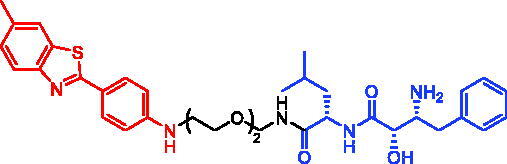	104
SNIPER-49	mHtt	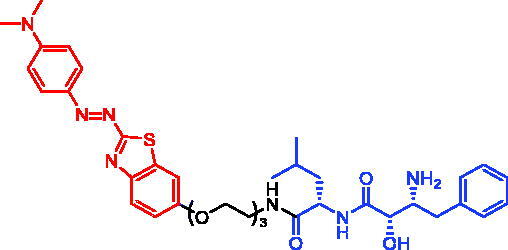	104

### 5.2. Targeting PDE4

Phosphodiesterases (PDE) are a diverse family of enzymes responsible for the degradation of cyclic adenosine monophosphate (cAMP) and cyclic guanosine monophosphate (cGMP) which are involved in several cellular and biochemical functions. Phosphodiesterase 4 (PDE4) is the major isoform within this group and is highly expressed in the mammalian brain. An inverse association between PDE4 and cAMP levels is the key mechanism in various pathophysiological conditions like airway inflammatory diseases-chronic obstruction pulmonary disease (COPD), asthma, psoriasis, rheumatoid arthritis, and neurological disorders. Due to the adverse effects like unbearable nausea and vomiting, dose intolerance and diarrhoea, PDE4 inhibitors have very less clinical compliance. Efforts are being made to develop PDE4 PROTACs having better efficacy and lesser adverse effects^105^^,^^106^.

In 2021, Mikihiko Naito et al. developed the discovery of SNIPER-50 (Table 20) against therapeutic target protein, PDE4, by conjugating the LCL161 derivative to a PDE4 inhibitor^107^. SNIPER-50 showed efficient protein knockdown activity against PDE4 protein at nanomolar concentration. The authors did not describe the blood–brain barrier permeability of SNIPER-50.

**Table 20.** Representative SNIPER targeting PDE4.

Compounds	Target protein	Structure	Reference
SNIPER-50	PDE4	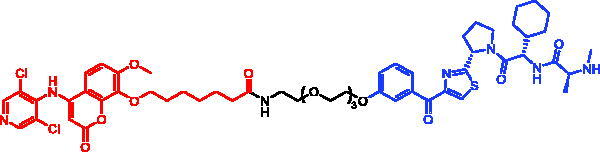	107

## 6. Snipers for others

### 6.1. Targeting RARα

Nuclear receptors (NRs), which are activated by small-molecular ligands, are transcription factors that regulate gene expression in the nucleus and play key roles in biological functions such as embryonic development and homeostasis. In addition, NRs are targets for the treatment of various diseases. Among NRs, retinoic acid receptors (RARα, RARβ, and RARγ) are receptors of all-trans retinoic acid (ATRA). Retinoids modulate the growth and differentiation of a wide variety of normal and transformed cells, and ATRA is involved in the control of embryonic development and cell differentiation^108^^,^^109^.

To achieve selective degradation for RARs, Yuichi Hashimoto et al. designed and synthesised SNIPER-51 (Table 21) by using Ch55 as ligand for RAR and bestatin as ligand for E3 ligase^72^. Western blot analysis showed that SNIPER-51 could downregulate RARα levels in a concentration-dependent manner in HT1080 cells expressing FLAG-tagged cIAP1, but exhibited no effect on the amount of CRABP-II, which suggested that SNIPER-51 showed selectivity for RAR knockdown activity against target protein at nanomolar concentration.

**Table 21.** Representative SNIPER targeting RAR.

Compounds	Target protein	Structure	Reference
SNIPER-51	RARα	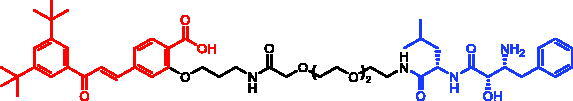	72

## 7. Conclusion

In this review, we provide a comprehensive update on the latest research progress in protein degraders that induce IAP-mediated ubiquitination. SNIPERs have emerged as a novel small molecule-based therapeutic approach in the treatment of many diseases, employing UPS to induce selective degradation of target proteins by hijacking E3 ligases, representing a very promising new paradigm for drug discovery. Compared to small-molecule drugs, SNIPERs display many features: (1) improving selectivities and specificities; (2) overcoming drug resistances; (3) eliminating the enzymatic and non-enzymatic functions of kinases; (4) degrading "non-pharmacological" protein targets; (5) rapid and reversible knockdown of POIs. Mikihiko Naito et al. reported the first small molecule that induces IAP E3-mediated ubiquitination and degradation of a target protein. Both selective knockdown of the target protein, and double knockdown of the target protein/cIAP1 were achieved by changing the chemical structure of the cIAP1 ligand. Indeed, double-proteins knockdown of IAP and cancer-related proteins is a promising approach for cancer treatment. This is a major advantage over other PROTACs that utilise the ubiquitin ligases CRBN and VHL. SNIPERs have performed well not only in cancer diseases but also in immune diseases, and neurodegenerative diseases. In recent years, more than 20 proteins^9^^,^^10^, including undruggable targets, can be degraded by SNIPERs.

Although SNIPERs depict a promising technology for a variety of aspects, including drug discovery and answers to biological issues, challenges exist for SNIPERs in the future. First, most of them have a high molecular weight and do not meet the requirements of the classical Lipinski's "Rule of Five." Second, linkers are also critical for SNIPERs, including membrane permeability and metabolic stability. To date, the principles guiding linker design, including composition and length, have not been rigorously mastered. There is an urgent need to do a lot of work to obtain the best linkers. Third, the mechanism of SNIPERs is not well studied, so more practices are needed to evaluate the absorption, distribution, metabolism, excretion, and toxicity of SNIPERs. Although SNIPERs face many challenges that need to be solved, they are potential to be developed as therapeutic agents for many incurable diseases.
